# Comprehensive Genome Analysis of 6,000 USA SARS-CoV-2 Isolates Reveals Haplotype Signatures and Localized Transmission Patterns by State and by Country

**DOI:** 10.3389/fmicb.2020.573430

**Published:** 2020-09-03

**Authors:** Lishuang Shen, Jennifer Dien Bard, Jaclyn A. Biegel, Alexander R. Judkins, Xiaowu Gai

**Affiliations:** ^1^Department of Pathology and Laboratory Medicine, Children’s Hospital Los Angeles, Los Angeles, CA, United States; ^2^Department of Pathology, Keck School of Medicine, University of Southern California, Los Angeles, CA, United States

**Keywords:** COVID-19, SARS-CoV-2, haplotype, variant, localized outbreak, temporal transmission

## Abstract

Genomic analysis of SARS-CoV-2 sequences is crucial in determining the effectiveness of prudent safer at home measures in the United States (US). By haplotype analysis of 6,356 US isolates, we identified a pattern of strongly localized outbreaks at the city-, state-, and country-levels, and temporal transmissions. This points to the effectiveness of existing travel restriction policies and public health measures in reducing the transmission of SARS-CoV-2.

## Introduction

SARS-CoV-2 is a positive-sense single-stranded RNA virus (Wu et al., 2020; Zhu et al., 2020). The collection of variants in a viral genome is referred to as the haplotype. New haplotypes arise from sequential acquisition of new variants in the viral genome. A haplotype, more than individual variants, serves as the distinct signature of a viral isolate and can be used effectively to trace the lineage, determine the ancestral origin of the infection, and to understand the community spread pattern during the COVID-19 pandemic. The rapid accumulation and sharing of SARS-CoV-2 genome sequences at an unprecedented speed have greatly facilitated haplotype-based genomic epidemiology studies. Since the first SARS-CoV-2 genome sequence was reported in January of 2020, as of May 20th 2020, there have been over 30,000 sequences deposited to GISAID (Elbe and Buckland-Merrett, 2017; Shu and McCauley, 2017),^1^ NCBI Virus,^2^ the China National Center for Bioinformation (CNCB) 2019 nCoV Resource (Zhao et al., 2020)^3^ and other data repositories (Shen et al., 2020b).

To understand the genetic etiology of COVID-19, it is imperative to have a comprehensive understanding of the variant and haplotype landscapes of all reported genomes of SARS-COV-2. Country-, state- and possibly city-specific variant profiles may contribute to varied disease exemplifications and fatality rates observed across the globe along with host factors such as age, ethnicity and comorbidity. In our previous study, we established a comprehensive COVID-19 genomic resource, Children’s Hospital Los Angeles (CHLA) COVID-19 Analysis Research Database (CARD), by harmonizing data from GISAID, NCBI Virus, CNCB and other resources (Shen et al., 2020b). Leveraging this extensive resource, in this study, we performed a comprehensive study of all publicly available SARS-CoV-2 genome sequences at the time of study, restricted to comparisons of global vs. USA isolates, which included 83 isolated that we sequenced at the Children’s Hospital Los Angeles (Shen et al., 2020b). We called variants from each genome sequence, and performed categorical analyses of variants and haplotypes as stratified by the geographic locations. This genomic epidemiology study, focusing on haplotypes, allowed us to gain insights as to the continuous evolution of the SARS-CoV-2 viral genomes and how the travel restrictions and safer-at-home health measures had been effective in reducing the spread of this pandemic by preventing the inter-state transmission of the virus.

## Materials and Methods

### Global and US SARS-CoV-2 Sequence Data

The CHLA internal SARS-CoV-2 sequencing data were generated using the SARS-CoV-2 whole genome sequencing research assay, established by the CHLA Center for Personalized Medicine and the Virology Laboratory. The major external resources of SARS-CoV-2 strains, genome sequences, and variants were GISAID, GenBank, CNCB, and NextStrain. Details about how the sequences were collected and further processed in CHLA COVID-19 Analysis Research Database (CARD) were described previously (Shen et al., 2020b).

### Sequence Alignment, Variant Calling, and Haplotype Analysis

Viral genome comparison and variant calling is a wraparound of MUMmer version 4.0.12 (Marçais et al., 2018),^4^ with results loaded into a MySQL database. Haplotype analysis was done with SQL queries and custom scripts, all as part of the CHLA COVID-19 Analysis Research Database (CARD) which was described previously (Shen et al., 2020b).

## Results

Viral genomes and demographic meta-data of 6,356 SARS-CoV-2 isolates within the US (as of May 20, 2020) were extracted from GISAID,^5^ GenBank,^6^ and COVID-19 patients and staff at the Children’s Hospital Los Angeles (CHLA). Variants, haplotype, geographic location at diagnosis and documented exposure for the patients were analyzed (Shen et al., 2020b). A total of 921 unique variants were each detected in at least three US isolates. Similarly, 264 distinct haplotypes were each represented in at least five US isolates (Supplementary Table S1). These variants and haplotypes were hence deemed unlikely to be sequencing artifacts and kept for further analysis.

The four most common mutations (241-C-T, 3037-C-T, 14408-C-T, 23403-A-G) were each present in about 65–67% of US isolates. In total, these 921 variants included 487 missense, 348 synonymous, 66 intergenic, 4 in-frame deletions, 5 stop gained/lost, and several other non-coding variants (Supplementary Table S2).

### US-Specific Haplotypes

**FIGURE 1:**
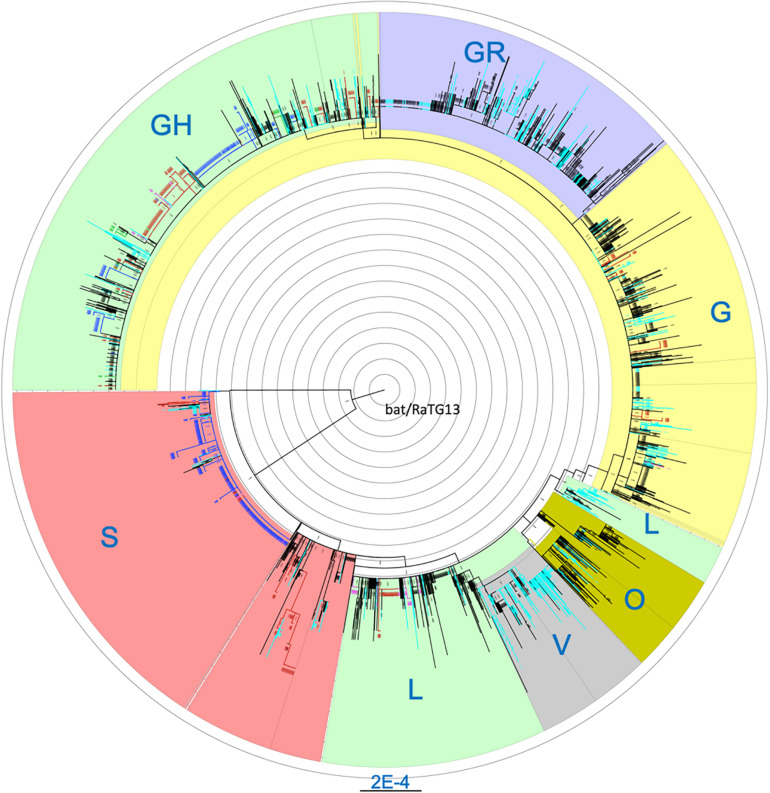
Maximum likelihood phylogenetic tree of isolates carrying US-specific haplotypes and 2,000 randomly selected non-US isolates. Blue, isolates with Washington-specific haplotypes; Pink, Children’s Hospital Los Angeles (CHLA) and other California isolates; Green, New York-specific, Red, other states’ isolates with USA-specific haplotypes; Light blue, UK and Australia isolates; Black, Other non-US isolates. Blue labels, the clade names. A total of 1107 isolates from US-specific haplotypes were included in the phylogenetic analysis. Non-US isolates belonging to any haplotypes were randomly sampled at about 10% to reach 2000 isolates. Each branch in the phylogenetic tree may represent a group of isolates. The tree is rooted at the outgroup MN996532 (EPI_ISL_402131, bat/Yunnan/RaTG13/2013).

Cross-stratification by geolocation identified city-, state- and country-specific haplotypes. Seventy seven of the 264 (29.2%) haplotypes that were found in at least five US isolates were US-specific. They comprise of a total of 849 isolates which accounted for 13.3% of the 6,356 US isolates. In addition to the 77 purely US-specific haplotypes, there were an additional four large haplotypes that were mostly North America-specific, with a total of 434 isolates where 431 isolates (99.3%) were from the US (425) and Canada (6) (Supplementary Table S3). Isolates from these four large haplotypes were geographically dispersed across the nation. Of note, all 66 US isolates belonging to haplotype (241-C-T, 1059-C-T, 3037-C-T, 11916-C-T, 14408-C-T, 18998-C-T, 23403-A-G, 25563-G-T, 29540-G-A) were from the COVID-19 epicenter in New York and neighboring states. Comprehensive phylogenetic analysis of the US-specific isolates, along with 2,000 randomly selected non-US isolates, revealed that the isolates fell exclusively in some major clades and were completely absent in the remaining clades (Figure 1). The mean number of isolates represented by each USA-specific haplotype was 11.4 ± 12.9 (range: 5–91) (Supplementary Table S3). Of note, 58 of the purely US-specific and the four nearly US-specific haplotypes consisted of 715 US isolates all had the globally dominant 23403-A-G (D614G) mutation (Korber et al., 2020). The 8782-C-T (orf1ab, synonymous) and 28144-T-C (orf8:p.Leu84Ser) variants were mutually exclusive with D614G, and co-occurred in 25 haplotypes that accounted for a total of 551 US isolates.

### State-Specific Haplotypes

State-specific haplotypes were identified for 12 states based on the sequences of 613 out of 6,356 US isolates (9.6%). Further, seven US-specific haplotypes were almost exclusively found in isolates from a single state, where the few exclusivity-violating isolates were from neighboring states. Washington state had the most private haplotypes (24 haplotypes and 367 isolates), followed by California (9 haplotypes and 63 isolates) and Utah (5 haplotypes and 44 isolates). The number of haplotypes increased over time as new variants were continuously acquired, but the newly emerged haplotypes were confined within these states to accumulate such that the percentage of isolates that carry the USA- and state-private variants and haplotypes increase (Supplementary Table S4). Two of the California specific haplotypes are notable. The 9-isolate 491-G-A,14940-A-G haplotype group and its single-marker ancestral haplotype (14940-A-G with 5 isolates) were exclusively present in California between March 31, 2020 and May 1, 2020. 491-G-A is a missense variant, p.Ala76Thr, in the orf1ab gene. The 15-isolate 25692-C-T haplotype group is similarly interesting in the sense that these haplotypes are relatively “ancestral” with only 1-base difference from the reference isolate genome (NC_045512.2), but they were recently seen in late April, after the inception of the safer at home policy in California. This is in contrast with the dominant haplotypes in the US that were more distant descendants of NC_045512.2, with at least 3 and frequently more than 10 variants compared to the reference. This is suggestive of containment of early infections in California and limited spread to other states, likely again because of the early response to the pandemic from the state of California.

On the national level, one major haplotype (8782-C-T, 17747-C-T, 17858-A-G, 18060-C-T, 28144-T-C) had 317 member isolates, where 315 (99.4%) were from the US (311, 98.0%) and neighboring Canada locations (4, 1.3%). With the exception of two isolates from Australia, there were no isolates from outside North America. It is noted that this haplotype lacks the dominant D614G mutation prevalent in Europe. The first reported USA COVID-19 case in mid-January, haplotype (3 variants 8782-C-T, 18060-C-T, 28144-T-C), is the more remote ancestral haplotype. Three cases from Washington collected around January 18 shared this 3-variant haplotype. The isolates were continuously present from late February through April 2020, with predominance found in the western states, including Washington and California, compared with isolates from the east coast. The potential immediate ancestral haplotypes inferred with CHLA CARD Genome Tracker were also from US and Canada isolates but they were sampled at later dates (Shen et al., 2020b). This provided further evidence of reduced state-to-state and coast-to-coast transmissions within the United States.

## Discussion

Based on the genomic analysis of all published SARS-CoV-2 sequences to date, safer-at-home measures have been very effective at reducing the spread of SARS-CoV-2, especially in preventing inter-state transmission of this highly infectious virus. The most state-private haplotypes were seen in the states of Washington and California where the earliest COVID-19 cases were reported and early safer-at-home orders were implemented. Persistent implementation of these measures would clearly lead to reduced spread of the COVID-19 pandemic over time. Indeed, in our recent re-analysis of SARS-CoV-2 genome data (August 3rd, 2020), we identified consistent, and even more prominent trend of localized haplotype patterns in California and other states. As an example, 3,048 of the 3,492 (87.3%) isolates from California carried state-specific haplotypes not seen in other states of USA.

On the other hand, a virus with a novel haplotype is not necessarily a new strain of the virus. The viral variant and haplotype analysis described here may prove to be critical, however, if a more transmissible and more deadly strain of SARS-CoV-2 emerges over time. Further studies will likely determine viral haplotypes, in the context of host factors, that may be associated with disease severity, response to treatment, or utility of vaccines for disease prevention.

## Author’s Note

This manuscript has been released as a preprint at MedRxiv (Shen et al., 2020a).

## Data Availability Statement

Publicly available datasets were analyzed in this study. This data can be found here: https://covid19.cpmbiodev.net/covid19/index.php.

## Author Contributions

All authors made major contributions to the conceptualization of the study, data interpretation, as well as the development of the manuscript.

## Conflict of Interest

The authors declare that the research was conducted in the absence of any commercial or financial relationships that could be construed as a potential conflict of interest.

## Footnotes

1 https://www.gisaid.org/

2 https://www.ncbi.nlm.nih.gov/labs/virus/vssi/#/

3 https://bigd.big.ac.cn/ncov/

4 https://mummer4.github.io/

5 https://www.gisaid.org/

6 https://www.ncbi.nlm.nih.gov/genbank/sars-cov-2-seqs/

## References

[B1] Elbe S. Buckland-Merrett G. (2017). Data, disease and diplomacy: GISAID’s innovative contributions to global health. *Glob. Chall.* 1 33–46. 10.1002/gch2.1018 31565258 PMC6607375

[B2] Korber B. Fischer W. M. Gnanakaran S. Yoon H. Theiler J. Abfalterer W. (2020). Spike mutation pipeline reveals the emergence of a more transmissible form of SARS-CoV-2. *bioRxiv* [Preprint], 10.1101/2020.04.29.069054

[B3] Marçais G. Delcher A. L. Phillippy A. M. Coston R. Salzberg S. L. Zimin A. (2018). MUMmer4: a fast and versatile genome alignment system. *PLoS Comput. Biol.* 14:e1005944. 10.1371/journal.pcbi.1005944 29373581 PMC5802927

[B4] Shen L. Dien Bard J. Biegel J. A. Judkins A. R. Gai X. (2020a). Comprehensive genome analysis of 6,000 USA SARS-CoV-2 isolates reveals haplotype signatures and localized transmission patterns by state and by country. *medRxiv* [Preprint], 10.1011/2020.05.23.20110452 PMC7509426 33013809

[B5] Shen L. Maglinte D. Ostrow D. Pandey U. Bootwalla M. Ryutov A. (2020b). Children’s hospital los angeles COVID-19 analysis research database (CARD) - A resource for rapid SARS-CoV-2 genome identification using interactive online phylogenetic tools. *bioRxiv* [Preprint], 10.1101/2020.05.11.089763

[B6] Shu Y. McCauley J. (2017). GISAID: global initiative on sharing all influenza data - from vision to reality. *Euro. Surveill.* 22:30494. 10.2807/1560-7917.ES.2017.22.13.30494 28382917 PMC5388101

[B7] Wu F. Zhao S. Yu B. Chen Y.-M. Wang W. Song Z.-G. (2020). A new coronavirus associated with human respiratory disease in China. *Nature* 579 265–269. 10.1038/s41586-020-2008-3 32015508 PMC7094943

[B8] Zhao W. M. Song S. H. Chen M. L. Zou D. Ma L. N. Ma Y. K. (2020). The 2019 novel coronavirus resource. *Yi Chuan.* 42 212–221. 10.16288/j.yczz.20-030 32102777

[B9] Zhu N. Zhang D. Wang W. Li X. Yang B. Song J. (2020). Novel Coronavirus from Patients with Pneumonia in China, 2019. *N. Engl. J. Med.* 382 727–733. 10.1056/NEJMoa2001017 31978945 PMC7092803

